# A breakthrough in liver regeneration for treatment of liver cancer

**DOI:** 10.20892/j.issn.2095-3941.2021.0293

**Published:** 2021-08-15

**Authors:** Hao Xu, Yisheng Pan, Ning Zhang

**Affiliations:** 1 Division of General Surgery, Peking University, Peking University First Hospital, Beijing 100034, China; 2 Translational Cancer Research Center, Peking University, Peking University First Hospital, Beijing 100034, China

The liver is the largest solid organ, and it is involved in multiple biological processes, including energy metabolism, protein synthesis, and detoxification^1^. Under physiological or pathological conditions, the liver can regrow to normal size even after resection of 90% of the liver volume because of its strong regeneration ability^2^. Changes in diet, viral infection (HBV and/or HCV), and cirrhosis are predisposing factors for hepatocyte damage and liver dysfunction. Patients with acute alcohol-associated hepatitis have a decreased number of hepatocytes around the central vein^3^. After HBV infection, activated immune cells attack the infected hepatocytes, resulting in extensive liver destruction. In nonalcoholic steatohepatitis, fat deposits in the liver and persistent chronic inflammation results in the destruction of liver cells, eventually leading to liver cirrhosis^4^. Most liver diseases are characterized by hepatocyte damage and decompensation of liver regeneration, which may eventually develop into liver cancer. Solving the decompensation of liver regeneration will be of great significance to the treatment of liver cancer. However, the subtype of cells that drives liver regeneration remains debatable.

Single-cell RNA sequencing of hepatocytes shows heterogeneity of hepatocytes in different zones^5^, although the exact cells involved in liver regeneration remain controversial. Certain stem-related genes have been identified as markers of hepatocytes with strong regeneration abilities. Chen et al.^6^ reported that hepatocytes, rather than a rare stem cell-like population, contributed to maintaining homeostasis. Other studies proposed that hepatocytes in specific locations that express specific markers may be the main cell population responsible for maintaining liver homeostasis. For example, Font-Burgada et al.^7^ found that hepatocytes located in the portal triads of the liver that expressed bile duct-enriched genes had a strong potential to regenerate under conditions of chronic liver injury. Wang et al.^8^ conducted lineage tracing and suggested that pericentral hepatocytes expressing the marker Axin2 were the main proliferating cell population in the uninjured liver. Sun et al.^9^ indicated that hepatocytes throughout the whole liver upregulated the expression of Axin2 and LGR5 in liver injury models without increasing the number of pericentral Axin2 (+) hepatocytes. Another study demonstrated that hepatocytes with high telomerase expression were the main cell population necessary for regeneration, both under conditions of homeostasis and in a damaged state^10^. These conflicting conclusions were derived from mice with different cell markers; therefore, the involvement of other unlabeled genes in liver regeneration could not be eliminated. Because we lack tools to label different cell populations in the liver, a clear conclusion remains elusive.

Cellular organization in the human liver is based on the building blocks of the hexagonal hepatic lobule. The lobule consists of portal triads, cords of hepatocytes, and central veins. Portal triads are comprised of a hepatic artery, a portal vein, and a bile duct, which radiate outward from the central vein and are sandwiched between a capillary network and a central draining hepatic vein. Hepatocytes are arranged in linear cords, through which blood drains towards the central veins. Historically, hepatocyte cords are divided into 3 zones according to physiological function (**Figure 1**). To identify the zone responsible for liver regeneration, Wei et al.^11^ generated a panel of 11 CreER knock-in mouse models combined with 3 pre-existing models for labeling cell subpopulations across the liver lobules, which enabled assessment of the function of different zones involved in liver maintenance and regeneration. The lineage tracing mouse models used in this study were generated by crossing the 11 gene-CreER knock-in mice with tdTomato fluorescent reporter mice, in which tamoxifen activated the expression of the red fluorescent protein at 6–8 weeks to track the proliferation of cell lineages at different time points. The results demonstrated that under homeostatic conditions, periportal zone 1 hepatocytes gradually contracted; midlobular zone 2 cells continued to proliferate and divide; zone 3 cells near the central vein (CV) did not repopulate; and biliary and other cellular compartments did not contribute to hepatocyte regeneration. These findings suggested that zone 2 was the main zone involved in liver repopulation under homeostatic conditions. In contrast to previous results^8^, researchers suggested that the Hamp (+) hepatocytes in zone 2 were the main cell subpopulation involved in liver regeneration during liver homeostasis. To identify the zone responsible for liver regeneration in a liver injury model, DDC and CCl_4_ were administered to induce liver injury in different zones. The results showed that hepatocytes in zone 2 played a critical role in liver regeneration in response to zone 1 damage caused by DDC or zone 3 injury induced by CCl_4_. To investigate the mechanism of regeneration in zone 2, they performed *in vivo* CRISPR knockout and activation library screening, and found that CCND1 was extensively expressed in zone 2. Moreover, the IGFBP2-mTOR-CCND1 axis was required for the preferential regeneration of zone 2 hepatocytes. The IGFBP2 acted on mTOR to regulate the expression of CCND1 in zone 2. In conclusion, they proved that zone 2 was the main zone involved in liver regeneration, regardless of the conditions of homeostasis or injury, and this process was regulated by the IGFBP2-mTOR-CCND1 pathway (**Figure 1**).

**Figure 1:**
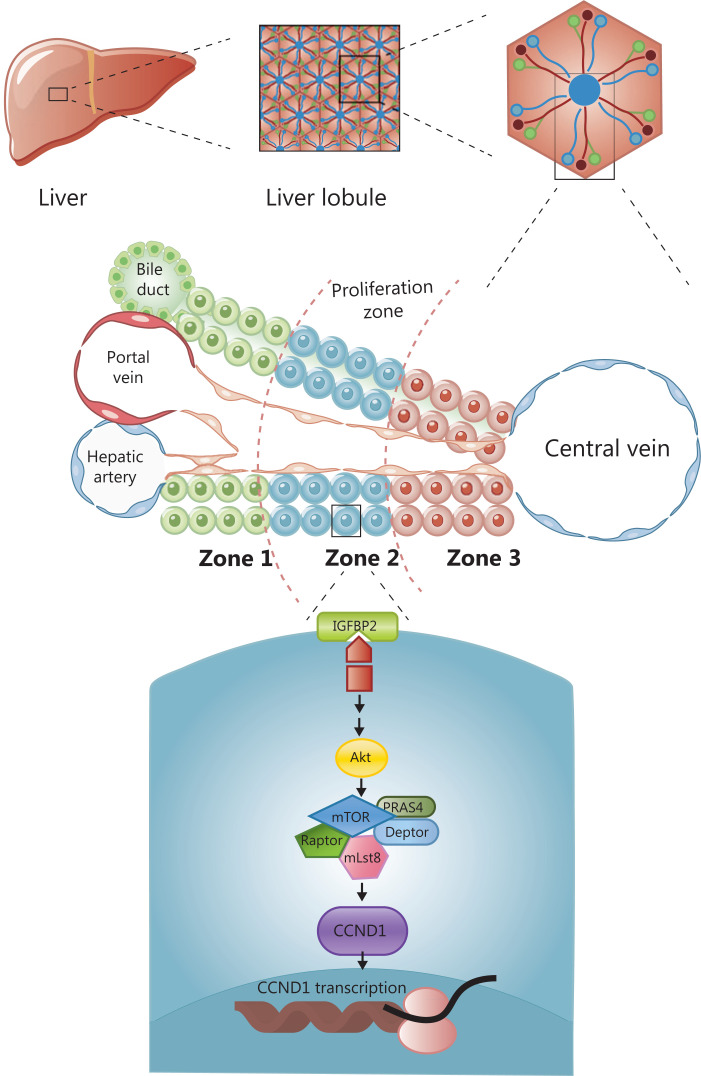
The division of liver lobules and the molecular mechanism of regeneration in zone 2. The lobule consists of portal triads, cords of hepatocytes, and central veins. Hepatocyte cords are divided into zones according to physiological function. The main zone for maintaining liver regeneration is zone 2, which is regulated by the IGFBP-mTOR-CCND1 pathway.

## Perspective

The groundbreaking advance of this latest work was the comprehensive labeling of multiple alleles on an unprecedented scale to trace the evolution of different lineages over a long period of time. The results showed that zone 2 was the main area responsible for maintaining liver regeneration under conditions of homeostasis and in a damaged state. Because previous studies showed that hepatocytes with a variety of highly expressed genes had the potential to regenerate, it was difficult to categorize the cells that maintained liver regeneration at the genetic level. The study complemented previous research and provided a relatively comprehensive conclusion. Moreover, the method used in the study for marking liver zones with multiple alleles could also be used for investigating the pathophysiological state of different zones under tumorigenesis.

Another contribution was the finding that proliferation of zone 2 hepatocytes was regulated by the IGFBP-mTOR-CCND1 pathway. Most liver diseases showed damage of hepatocytes and decompensation of liver regeneration, which may eventually develop into liver cancer. This discovery has important translational implications for the design of treatment strategies for liver cancer. Liver transplantation, which is the most effective treatment for liver cancer, is not available for many patients because of the limited number of donors. Therefore, *in vitro* culture of hepatocytes has become the focus of liver research aimed at overcoming the deficiency of liver donors^12^. Although the factors that affect liver regeneration have been previously investigated^13,14^, there is no study characterizing related signaling pathways. The results of this study provided theoretical support for the *in vitro* culture of hepatocytes, especially for the *in vitro* culture of autologous hepatocytes, and may greatly improve the efficacy of hepatocyte culture for therapeutic purposes, which will shed light on possible treatments of liver cancer.

The study had several limitations. Although the alleles used in the study were comprehensive, the analysis was not specific to each zone. Most of the genes analyzed were expressed in every zone at different expression levels. Identifying markers expressed only in specific zones is important. The IGFBP-mTOR-CCND1 pathway regulated proliferation in zone 2 under conditions of homeostasis. However, whether it was still the main pathway regulating proliferation under conditions of liver damage was not verified. In addition, dynamic proliferation was not assessed in this study and should be considered in future analyses^15^. Overall, this study characterized midlobular zone 2 hepatocytes and elucidated their role in regeneration under homeostatic and injured liver conditions.
